# Pediatric Off-Label COVID-19 Vaccination: The Concerns of Healthcare Professionals in Pakistan

**DOI:** 10.3390/vaccines10081236

**Published:** 2022-08-02

**Authors:** Sadia Shakeel, Shagufta Nesar, Ghazala Noor Nizami, Zafar Iqbal, Shaista Emad, Quratulain Wasim, Tayyaba Mumtaz, Shazia Jamshed, Muhammad Salahuddin Usmani, Rabia Hussain

**Affiliations:** 1 Department of Pharmacy Practice, Faculty of Pharmaceutical Sciences, Dow College of Pharmacy, Dow University of Health Sciences, Karachi 74200, Pakistan; 2 Jinnah College of Pharmacy, Sohail University, Karachi 74000, Pakistan; shaguftausmani@sohailuniversity.edu.pk (S.N.); tayyabafaraz786@gmail.com (T.M.); 3 Jinnah College of Rehabilitation Sciences, Sohail University, Karachi 74000, Pakistan; ghazala.noor.n@gmail.com; 4 Jinnah College of Nursing, Sohail University, Karachi 74000, Pakistan; zi.iqbal@yahoo.com; 5 Department of Biochemistry, Jinnah Medical & Dental College, Sohail University, Karachi 74000, Pakistan; shaista.emad@yahoo.com; 6 Department of Pharmacy Practice, Faculty of Pharmacy, Jinnah University for Women, Karachi 74600, Pakistan; ainakhan1@outlook.com; 7 Department of Clinical Pharmacy and Practice, Faculty of Pharmacy, Universiti Sultan Zainal Abidin, UniSZA, Kuala Terengganu 21300, Malaysia; shaziajamshed@unisza.edu.my; 8 Sindh Medical College, Jinnah Sindh Medical University, Karachi 75510, Pakistan; salahuddin_usmani@outlook.com; 9 Discipline of Social and Administrative Pharmacy, School of Pharmaceutical Sciences, Universiti Sains Malaysia, Pulau Pinang 11800, Malaysia

**Keywords:** COVID-19 vaccinations, off-label use, healthcare professionals, Pakistan

## Abstract

Global health authorities have emphasized the vital role of healthcare professionals (HCPs) as a reliable source of vaccination information for patients in primary care. However, HCPs are concerned whether COVID-19 vaccinations can be used off-label. Hence, the current study was conducted to assess their perspectives towards off-label COVID-19 immunization in children. The study tool, consisting of 40 items, was utilized to evaluate HCPs’ knowledge and attitudes towards the off-label use of the COVID-19 vaccine in children under 12 years of age. To assess the unfavorable attitudes regarding vaccinations, the Vaccination Attitudes Examination Scale was employed. Overall, 477 completed questionnaires were incorporated in the present study, with a response rate of 88.9%. The mean age of the respondents was 38.6 ± 7.5 years; among whom the majority were physicians, *n* = 209 (43.8%), and pharmacists, *n* = 112 (23.4%). Approximately 78% of the respondents had a general awareness of off-label vaccination. Around 80% knew the adverse drug reactions associated with the use of COVID-19 vaccines. Females showed more mistrust about vaccine benefits, *n* = 55 (16.9%), compared to males, *n* = 21 (13.8%), and concerns about commercial profits of vaccines, *n* = 59 (18.1%), compared to males, *n* = 19 (12.5%). By profession, physicians showed statistically significantly lower mistrust, *n* = 18 (8.6%), and higher concerns about unpredicted effects of vaccines, *n* = 41 (19.6%). A major portion of the respondents, *n* = 327 (68.5%), did not consider that HCPs should prescribe/administer off-label COVID-19 vaccination in children. The current findings demonstrated that respondents had an appropriate level of understanding about COVID-19 immunization in children. They showed higher levels of rejection for off-label use of the COVID-19 vaccination.

## 1. Introduction

The coronavirus disease (COVID-19) arose in China and spread all over the world. Based on reports of the disease’s prevalence and rapid global spread, on 30 January 2020, the World Health Organization (WHO)’s Emergency Committee declared it a Public Health Emergency of International Concern (PHEIC) [1]. COVID-19 has impacted many people worldwide in a short time and caused severe complications in pregnant women, the elderly and individuals with chronic debilitating disorders [2]. Children were certainly impacted during the pandemic. Special precautions were taken to prevent COVID-19 infection in this age group due to the weakening of their immune system, particularly in newborns [3]. Hospitalization rates for children with COVID-19 are much lower than those for adults, indicating that children may have less severe disease than adults [4]. However, the true frequency of COVID-19 infection in children is unknown due to a lack of extensive testing and a focus on adults and those with severe disease. It is crucial to highlight that children infected with SARS-CoV-2 might present with other dangerous illnesses, such as diabetic ketoacidosis or intussusception, and that a broad differential perspective must be maintained while evaluating unwell children during the COVID-19 pandemic [5].

Immunization of people is a leading concern of public health in a worldwide pandemic environment. The effectiveness of COVID-19 immunization is hugely reliant on the population’s trust in vaccination [6]. Various trials have demonstrated the efficacy of the vaccines available in the market, with Pfizer, Moderna, and Sputnik V reporting efficacy levels exceeding 90%, and AstraZeneca and Janssen reporting efficacy levels above 60% [7]. The Pfizer-BioNTech COVID-19 vaccination for children aged 5 to 11 years received Emergency Use Authorization from the Food and Drug Administration on October 29. In children aged 5 to 11 years, the Pfizer-BioNTech COVID-19 vaccine has a high efficacy (>90%) against COVID-19, and the advantages of vaccination outweigh the hazards. Children must receive the COVID-19 vaccine to prevent the spread of SARS-CoV-2 in the community and to protect them against it [8]. However, the matter of whether all children under the age of 12 should be immunized against COVID-19 is still being debated. Because of the relatively modest risk posed in children by acute COVID-19, as well as ambiguity regarding the proportional effects of vaccination and illness, the risk-benefit balance of immunization in this range of age is more complicated [9]. The American Academy of Pediatrics (AAP) warned against using the vaccine off-label in children, and the Centers for Disease Control and Prevention (CDC)’s vaccine provider agreement likewise cautioned against it [10].

The first documented COVID-19 case was reported in Pakistan on 26 February 2020, in Karachi [11]. The Ministry of Health put in continuous effort and modified its policies in response to the COVID-19 outbreak so that adults, who account for nearly half of a population of 225 million, could receive a vaccine. The first shipment of Oxford-AstraZeneca COVID-19 vaccines (SII-AZ AZD1222) for Pakistan arrived from the COVAX facility with 1,238,400 doses of vaccine on 8 May 2021, followed by another shipment of 1,236,000 [12]. The National Deployment and Vaccination Plan, created under the direction of the National Command Operation Center (NCOC) Pakistan, initially prioritized older citizens, frontline health workers, and other priority groups that needed to be immunized [12]. According to the Drug Regulatory Authority of Pakistan (DRAP), two COVID-19 vaccines have received Emergency Use Authorization (EUA) under specific restrictions. Both vaccinations underwent quality and safety assessments. An AstraZeneca vaccine was given EUA on 15 January 2021. Another vaccine produced by China National Pharmaceutical Group (SinoPharm, Beijing, China) was also granted EUA in a meeting held by the Registration Board of DRAP in Pakistan on 18 January 2021 [12].

The Government of Pakistan’s efforts to ensure a smooth and successful rollout of the COVID-19 vaccination program have the support of United Nations Children’s Fund (UNICEF) [13]. In this regard, the government purchased COVID-19 vaccines, increased the cold chain capacity to assure safe vaccine storage and also took the lead in managing risk and community involvement to boost vaccination rates and assured adherence to COVID-19 safety precautions. The Ministry increased the Expanded Program on Immunization’s (EPI) current cold chain capacity, which now includes 15,000 cold freezers. Additionally, it has established 23 ultra-cold chain facilities in 15 major cities. In addition, it has developed procedures for the safe distribution of vaccines throughout the country, the safe and environmentally acceptable disposal of waste, including used syringes, and SMS registration for immunization [13]. The NCOC updated its COVID-19 immunization recommendations for people under the age of 18 on 1 September 2021; adding that individuals under the age of 18 will receive the Pfizer vaccine. On 28 September 2021, the Federal Minister for Planning, Development, and Special Initiatives revealed that the NCOC had chosen to begin immunizing children who were 12 years old [14]. According to the guidelines established by NCOC, children between the ages of 12 and 18 should only receive Pfizer, Sinopharm, and Sinovac vaccines. Furthermore, to make it easier for children to be vaccinated, a special vaccination campaign was held in all public and private schools [14]. Global authorities have emphasized the significant contribution of healthcare professionals (HCPs) in instilling trust in COVID-19 vaccinations [15]. The acknowledgement of the benefits of the COVID-19 vaccine by HCPs is significant in combating the pandemic because they have an influential impact on patient vaccination choices. HCPs could accomplish their responsibilities to debunk misconceptions, eliminate misinterpretation, and deliver accurate information about risks and benefits so that patients may make educated decisions. Physicians have prescribing authority for medicines and biologics that are ‘off label’, that is, they are used in ways other than those for which they were legally authorized [15]. Off-label drug use implies the fact that their safety and/or effectiveness were not reviewed or otherwise demonstrated through the Food and Drug Administration (FDA) review process for the conditions and diseases examined. Off-label use of drugs and biologics occurs when they are given for medical conditions other than those indications for which they were authorized, at a higher or lesser than specified doses, and in people other than those studied during the FDA approval process. DRAP is the regulatory authority for drug use in Pakistan, established in 2012, that aims to provide effective regulation of drugs and health products. However, the policies regarding off-label use of the drug are still in progress. Hence, Pakistan complies with both the 2007-implemented European Union Regulation on Medicinal Products for Pediatric Use (regulation n° 1901/2006) and the FDA recommendations for drug use [16]. HCPs have a strong devotion to practice standards and feel obligated to promote vaccinations. However, they are concerned whether COVID-19 vaccinations can be used off-label. As a result, it is important to assess their perspectives toward the off-label COVID-19 immunization in children under 12.

## 2. Materials and Methods

### 2.1. Study Design and Setting

A quantitative survey(Table S1) based cross-sectional study was conducted on HCPs, which includes physicians, dentists, pharmacists, nurses and other HCPs working in Karachi between September and December 2021. The study was carried out in Karachi, the capital of the Sindh province that has grown significantly over the past few decades. By 2030, Karachi is anticipated to rank as the seventh-largest metropolis in the world as a result of its rapid growth [11]. There are currently more than 14,000 doctors, over 10,000 registered pharmacists, 2000 nurses, and more than 12,000 paramedics working for the Sindh Department of Health throughout the province. The questionnaire was developed using Google Forms and was then disseminated via different social media platforms. Some of the respondents whose official email addresses were available were invited to participate through email. The respondents used their devices to access the URL and complete the questionnaire. Respondents were deemed eligible for the study provided they were registered with the appropriate professional body for accreditation and agreed to voluntarily participate in the study.

### 2.2. Sample Size Calculation

The sample size was calculated by Raosoft sample size calculator [17], which was found to be *n* = 377. However, in order to improve the response rate and to further generalize the findings, we increased the sample size by another 50% of the originally calculated sample size. Hence, 536 questionnaires were distributed among the potential respondents. The approach of convenience sampling was used for the study.

### 2.3. Survey Instrument

The study tool, consisting of 40 items, was developed in the English language after an extensive literature review of the previous studies published on the topic of the off-label use of COVID-19 vaccine in children under 12 years of age [9,10,18,19,20,21]. A team of experts, comprised of three general physicians and two clinical pharmacists from Sohail University, evaluated the questionnaire’s content validity. Key check and item response analysis was used to determine the tool’s construct validity. The questionnaire’s face validity was tested by administering it to a group of ten individuals who were not respondents in the research. Following a series of conversations with the group, the questionnaire was finalized. Internal consistency of the knowledge estimating element of the instrument was analyzed by the Kuder–Richardson formula 20 (K-R 20), which yielded a strong internal consistency of K-R 20 coefficient = 0.766, whereas Cronbach’s alpha = 0.802 for the attitude evaluating section. A test-retest correlation was performed to examine the tool’s stability. Pearson’s product-moment correlations of 0.868 (*p* < 0.001) and 0.850 (*p* < 0.001) indicated that the knowledge and attitude evaluation portions of the instrument were quite stable.

### 2.4. Tool Administration and Scoring

In addition to the consent to participate and demographic information, the questionnaire had 18 multiple-choice questions for knowledge evaluation, with the options of “true, false and do not know”. Every correct response scored a “1” point, while each incorrect answer, and do not know option and unsolved question received a “0.” A sufficient level of knowledge was determined as correctly answering 12 out of 18 questions (>60 percent). The WHO and CDC official websites were used to generate the questions and correct answers for the knowledge section of the questionnaire.

The Vaccination Attitudes Examination (VAX) Scale was used, which was rephrased in the context of the current study [21]. The respondents were advised to put emphasis on the specific scenario of COVID-19 vaccination in children under 12 when answering this 12-item assessment. Responses were graded on a 6-point scale from 1 “strongly agree” to 6 “strongly disagree”

The four sub-scales were as follows: (i) mistrust/trust of vaccine benefit (e.g., “I feel children are safe after being vaccinated with COVID-19 vaccine”), (ii) concerns about the unpredicted effect in future (e.g., “I am concern about the unknown effects of COVID-19 vaccine in the future”); (iii) commercial profit concerns (e.g., “Vaccines make a lot of money for pharmaceutical companies, but do not do much for general public”); and (iv) preference for natural immunity (e.g., “Natural immunity lasts longer than vaccination”), including 3 items in every domain. The four subscales and the questions for attitude toward vaccination were classified as having high (a score in between 5 and 6), moderate (a score in between 3 and 4), or low (a score in between 1 and 2) levels of unfavorable attitudes about vaccines.

Furthermore, there were questions that focused on the HCP’s attitude towards off-label COVID-19 vaccination in children and the sources of information of respondents for COVID-19.

### 2.5. Data Analysis

The baseline characteristics of the respondents were evaluated by descriptive statistics. The continuous data were represented as means and standard deviations (SD), whereas categorical variables were represented as %. The Shapiro–Wilk test was conducted to evaluate the normality distribution of the variables. Because of the non-normal distribution of the data, the Mann–Whitney U test and the Kruskal–Wallis test were used. The association between demographic features of the respondents and dependent variables was determined using linear regression statistical analysis. Gender and working organization were classified as dichotomous indicators, age as a continuous indicator, and experience as an interval scale. At the 95% confidence level, a statistically significant variance was considered at the *p*-value of <0.05. SPSS for Windows version 24.0.0 (SPSS, IBM Inc., Chicago, IL, USA) was employed for data analysis.

### 2.6. Ethical Approval

The study was approved by the Ethical Review Committee of Sohail University with the protocol # 000125/21 and written consent was taken from the respondents before the study.

## 3. Results

### 3.1. Demographic Characteristics

Overall, 477 completed questionnaires were included in the study, with a response rate of 88.9%. The mean age of the respondents was 38.6 ± 7.5 years; among whom 152 (31.8%) were males and *n* = 325 (68.1%) were females. Among the participating HCPs, the majority were physicians, *n* = 209 (43.8%), and pharmacists, *n* = 112 (23.4%). More than 70% of the respondents work in private sector organizations. Nearly half of the respondents work in primary patient care, and *n* = 188 (39.4%) had an experience of 1–5 years. Table 1 represents the complete demographic information of the respondents.

### 3.2. Respondents’ Knowledge towards Off-Label COVID-19 Vaccination in Children

Approximately 78% of the respondents had a general awareness of off-label vaccination (see Table 2). However, only *n* = 267 (55.9%) could provide an example (other than COVID-19 vaccine) that is used off-label. A linear regression statistical analysis depicted that the understanding of off-label vaccination statistically varied with the gender, age, profession, practice area and experience of respondents. The majority of the respondents, *n* = 385 (80.7%), knew the COVID-19 vaccines approved for used.

The respondents *n* = 359 (75.2%) knew the benefits of COVID-19 vaccine in children; whereas *n* = 316 (66.2%) knew that the COVID-19 vaccine is safe and effective. The respondents *n* = 302 (63.3%) knew that the FDA and the AAPs both caution against using the COVID-19 vaccine off-label in children. More than 60% respondents knew that children should get the age-appropriate vaccine formulation irrespective of their size or weight. The respondents *n*= 318 (66.6%) knew that the COVID-19 vaccine specified for adults and teens could not be administered to children aged 5 through 11.

Around 70% of the respondents in the present study knew that children can get other vaccines in combination with the COVID-19 vaccination in the same visit. Around 80% knew the adverse drug reactions of the COVID-19 vaccines. The respondents *n* = 346 (72.5%) knew that COVID-19 vaccines do not cause any developmental or fertility problems in vaccinated children.

### 3.3. Respondents’ Attitude towards Off-Label COVID-19 Vaccination in Children

Table 3 depicts the respondents’ attitudes toward COVID-19 vaccines (four areas of the VAX scale concerns about mistrust/trust of vaccine, unpredicted effects, commercial profits, preference for natural immunity and agreement with off-label COVID-19 vaccines in children under 12) by age, gender, profession, work organization, practice area and experience. The first variable of the demographic characteristics from each category presented in Table 1 was taken as a reference in Table 2 and Table 3.

By gender, females showed higher mistrust, *n* = 55 (16.9%), compared to men, *n* = 21 (13.8%), and concerns about commercial profits, *n* = 59 (18.1%), compared to men, *n* = 19 (12.5%). By profession, physicians presented statistically significantly lower mistrust, *n* = 18 (8.6%), and higher concerns about unpredicted effects, *n* = 41 (19.6%). By work organizations, those who were employed in public sector organizations presented a statistically significant higher inclination towards mistrust, *n* = 17 (13.1%), and preference to natural immunity, *n* = 23 (17.8%), as compared to those employed in private sector organizations (*n* = 38; 10.9% and *n* = 44; 12.6%, respectively). Those with less working experience were less concerned about the unpredicted effects, *n* = 11 (5.8%). The respondents *n*= 254 (53.2%) depicted highly negative attitudes in agreement with off-label COVID-19 vaccination in children under 12.

Table 4 represents the respondents’ perceptive towards off-label COVID-19 vaccination in children under 12. A major portion of the respondents, *n* = 327(68.5%), did not consider that HCPs should prescribe/administer pediatric off-label COVID-19 vaccines, with the responses varying significantly with their gender (*p* = 0.0001) and working organization (*p* = 0.003). More than half of those surveyed disagreed that pediatric off-label COVID-19 immunization is subject to the same legal and ethical standards as other cases of off-label usage; the response was associated significantly with their working organization (*p* = 0.007). The vast majority of the respondents, *n* = 435 (91.1%), agreed that emerging clinical studies will provide evidence on the most effective and safe dose and administration schedule for children. The major sources of respondent information was electronic media, including WHO, FDA and CDC websites *n* = 201 (42%), medical journals/literature, *n* = 105 (22%), and the ministry of health website, *n* = 96 (20%).

## 4. Discussion

Pakistan is ranked 154 out of 195 nations in the worldwide Healthcare Access and Quality (HAQ) index, where the burden of disease is high [11]. Thus, the spread of COVID-19 is distressing in this dire situation when the country’s health care system is already incapable of meeting the needs of its 208.8 million citizens [22]. Vaccine reluctance might be viewed as a global health risk. Whether children should be administered COVID-19 immunization has been contentious, particularly for those under 12 years old, because the risk-benefit ratio in this age range is more challenging [23]. The present study revealed that approximately 78% of the respondents had a general awareness of off-label vaccination. The variables that influence HCP’s roles in recommending the use of vaccines include their understanding of and trust in a vaccine, the accessibility of evidence-based information, and the chance to advocate vaccinations [24]. The majority of the respondents knew the vaccination benefits and stated that the COVID-19 vaccine is safe and effective for children aged 5 through 11. Pfizer’s COVID-19 vaccination for children is more than 90% successful to prevent COVID-19 in children aged 5 to 11 years [8]. The majority of the respondents knew that the FDA and the AAPs both were cautious against COVID-19 vaccine off-label use in children under 12. The COVID-19 vaccine provider agreement by CDC mandates practitioners to only administer this vaccine in accordance with FDA Emergency Use Authorization (EUA) [16]. Physicians who break this agreement may face legal consequences. It also means that physicians and patients seeking off-label use of COVID-19 vaccinations may be limiting patients’ ability to be compensated for vaccine injuries or applying lawsuits against physicians [12]. More than 60% of the respondents knew that children should get the age-appropriate formulation of vaccine, irrespective of their size or weight and that the COVID-19 vaccine specified for adults could not be given to children under 12. Unlike many medicines, vaccination doses are determined by age rather than weight or size. The dose must be determined by the age of the child on the day of immunization [25].

Around 70% of the respondents in the present study knew that children can get other vaccines in combination with COVID-19 vaccine in the same visit. Pakistan is still battling vaccine-preventable illnesses (VPD) [11]. Vaccination coverage in rural children is inadequate due to a variety of challenges, such as cost, hesitation, and a lack of understanding [26]. COVID-19 has reduced vaccination rates in Pakistan, owing to limited travel, vaccine shortages, and inadequate coverage. During the current epidemic, there is a substantial possibility that children may contract VPD, resulting in yet another infectious disease disaster [27]. This risk, however, may be mitigated if COVID-19 immunization is paired with the administration of other standard vaccinations [26]. Around 80% of the respondents knew that COVID-19 vaccinations, as with any other vaccine, may have unusual adverse effects. The development of myocarditis or pericarditis following mRNA vaccines has recently been a source of concern, especially in male adolescents [27]. Another study found 10.7 incidences per 100,000 people in males who were 16–29 years old. Approximately 6% of these individuals required critical care hospitalization. However, the vast majority of patients healed with no long-term consequences (86 percent had their symptoms resolved after a mean duration of 35 days) [28]. The major associations have been observed for myocarditis following mRNA-1273 vaccination in persons aged 18 to 24 years [29]. However, there is no relationship between COVID-19 vaccinations and long-term infertility in children or adults, according to experts [30].

A small portion of respondents consider COVID-19 vaccines safe for children under 12 years of age and the majority showed preference for natural immunity. Our study reported that physicians presented significantly lower mistrust and higher concerns about unpredicted effects of vaccines as compared to other HCPs. It is reported that despite favorable attitudes among healthcare workers for future COVID-19 vaccinations, vaccine hesitancy remains prevalent, particularly among nurses [31]. A survey conducted in Israel revealed considerable disparities in vaccination intentions amongst physicians, nurses, and the general public. Nurses (61%) had lower acceptability than the general public (75%) and doctors (78%) [32]. These findings are consistent with those of research carried out in the United States, where medical students and physicians were more willing to get vaccinated as compared to the nurses and patient care associates [33]. The present findings revealed that females had higher mistrust (16.9%) compared to men (13.8%) and concerns about commercial profits of vaccine (18.1%) compared to men (12.5%). Similar findings were reported by another study, depicting that the vaccine hesitancy was significantly associated with gender [34]. The Ministry of Health is combating the spread of false information in a nation that was already struggling with polio vaccination rejection in some places, with assistance from UNICEF [11]. When it comes to COVID-19, the hesitation is frequently based on misconceptions, such as the vaccine causing infertility, the pandemic being a foreign scheme, or the virus being benign. UNICEF conducted ethnographic surveys to identify the current views towards the pandemic and vaccination, digital media analysis, and a KAP survey to understand vaccine hesitancy to combat widespread misinformation. The findings assisted the Ministry in modifying its approach and the kind of information disseminated to the public via traditional and social media. Since last year, the Ministry has created and distributed materials such as leaflets, online flyers, etc. in the six national languages, including famous people, religious figures, important media outlets, and teenagers [13].

Studies have revealed that people who have been infected can significantly benefit from vaccination. It provides them a robust, long-lasting immunity boost [35]. Nearly half of the respondents think that children who are not immunized could have their social, emotional, and intellectual abilities hampered. The majority of the respondents did not support the off-label use of COVID-19 vaccination in children under 12. The approval of the vaccination for adults, but not for children, places doctors and pharmacists in an odd situation. Their professional commitment to act as gatekeepers—to refuse to prescribe a medicine or fulfill a prescription if they do not believe it is justified—seems to conflict with their professional aims of keeping patients safe and healthy [36]. The general public may not grasp the reasons for variations between the label and the recommendations if public health authorities do not give adequate explanations and communication to HCPs. This may reduce trust in public health recommendations and lead to vaccination hesitancy, lower adherence to immunization schedules, and/or public health-advised vaccine usage if used outside of the labeled purpose. More than half of those surveyed disagreed that pediatric off-label COVID-19 immunization is subject to the same legal and ethical standards as other cases of off-label usage. There are certain hindrances to encourage the use of off-label pediatric COVID-19 vaccines. The major fact is that it is not yet known which dosage is best for newborns and young children [8]. For this reason, the AAPs warned against off-label usage before the EUA for children aged 5–11 was released [37]. The vast majority of the respondents agreed that emerging clinical studies will provide evidence on the most effective and safe dose and administration schedule for children. Safety evidence is an essential part of each regulatory submission for a COVID-19 vaccine, which is gathered during the vaccine development process [7]. Thus, robust assessment of safety data from trials on vulnerable populations would provide confidence to HCPs in encouraging the use of vaccines in children.

This study adds on to the research that has been conducted to investigate HCP’s perspectives about off-label COVID-19 vaccination in children. They agreed that ‘off-label’ usage is prevalent in medicine; however, when it comes to COVID-19 vaccinations, the issues are a little more complex and HCPs should not prescribe/administer pediatric COVID-19 vaccines off-label. Moreover, off-label medicine prescriptions are far more common in the pediatric population of Pakistan. The responsibility of deciding when off-label medication is suitable for patients relies primarily on the medical profession. The physicians consider whether there is sufficient evidence to rationalize off-label use; they inquire for further information when sufficient evidence is required. However, it may be challenging for physicians and other HCPs to prescribe drugs appropriately for off-label uses due to time constraints, information overload, and the involvement of the industry in research and education about off-label drug usage. To reduce the likelihood of adverse medication events related to off-label drug use, evidence based on recent clinical research is required. Hence, there is a need for policy efforts to improve the quality of off-label prescribing [16].

The study’s limitations include the limited sample size and the fact that all surveys were conducted in a single city, Karachi. It might not be representative of other regions in Pakistan; however, Karachi, is the biggest city of Pakistan. Thus, it is expected that the findings from the other parts of the country would not be very different from it. In addition, the nature of the study was an online cross-sectional study and there may be selection bias, making it less likely to be representative of all HCPs practicing in Karachi. However, physicians dominate the Pakistani healthcare system as leading HCPs of society.

Another limitation of the study is that the respondents were not asked whether they had any experience caring for children. This might affect their decision to pay attention to the information on childhood vaccinations and their willingness to learn the facts about the questions that they were being asked. However, this study was conducted in the biggest city of the country, where the healthcare system is more developed and advanced. Thus, we believe that this study presents findings that could be considered useful by other HCPs, as well as policy makers, in the country.

## 5. Conclusions

The current findings demonstrated that respondents had an appropriate level of understanding about COVID-19 immunization in children. They had a relatively low unfavorable attitude towards vaccination benefit suspicion, the unpredictability of future effects, commercial profit concerns, and a preference for natural immunity. They showed higher levels of rejection for off-label use of COVID-19 vaccination. They agreed that mass COVID-19 immunization of all children under the age of 12 might become the worldwide standard in the future. However, it is important, at the moment, to consider the benefits and hazards with prudence and proceed with caution.

## Figures and Tables

**Table 1:** Characteristics of study population.

Baseline Characteristics	Frequency (%)
Age (years)	38.6 ± 7.5
Gender	
Male	152 (31.8)
Female	325 (68.1)
Profession	
Physicians	209 (43.8)
Pharmacists	112 (23.4)
Dentists	28 (5.8)
Nurses	96 (20.1)
Other HCPs	32 (6.7)
Working Organization	
Private sector	348 (72.9)
Public sector	129 (27)
Practice area	
Primary patient care	225 (47.1)
Secondary patient care	57 (11.9)
Tertiary patient care	195 (40.8)
Working Experience	
1–5 years	188 (39.4)
6–10 years	149 (31.2)
11–15 years	54 (11.3)
>15 years	86 (18)

**Table 2:** Respondents’ knowledge towards off-label COVID-19 vaccination in children.

Knowledge Item	Correct Response *n* (%)	Age	Gender	Profession	Work Organization	Practice Area	Work Experience
Off-label vaccination in children							
Definition/general understanding	369 (77.3)	0.019	0.014	0.012		<0.001	0.01
Any example (other than COVID-19 vaccine)	267 (55.9)			0.02			
COVID-19 vaccines							
Any example of vaccine	385 (80.7)			0.006	0.004		
Benefits	359 (75.2)		<0.001	0.003	<0.001	0.017	
Safety and efficacy	316 (66.2)	0.002			0.01		
COVID-19 vaccines in children							
FDA * and AAP ** caution for COVID-19 vaccine off-label use	302 (63.3)	0.005	0.008	0.003	0.001		
Administration of COVID-19 vaccines							
Age-appropriate vaccination	294 (61.6)	0.001	0.009	0.001	<0.001		
Formulation specification	255 (53.4)			<0.001	0.005	0.06	<0.001
Dosing specification	318 (66.6)		0.03				
Monitoring requirements	329 (68.9)			0.001			<0.001
Co-administration of other vaccines in the same visit	330 (69.1)		0.001				
Adverse drug reactions of COVID-19 vaccines							
Examples	379 (79.4)						
Severity of adverse reactions	377 (79)						
Developmental or fertility problem	346 (72.5)						
Risk for myocarditis and pericarditis	289 (60.5)	<0.001		0.016		0.02	
Post-vaccination scenarios							
Chances of getting COVID-19	326 (68.3)						
Chances of spreading COVID-19 to others	312 (65.4)				0.003		
Severity of disease if get COVID-19	386 (80.9)						

A *p*-value is a probability that is determined after data are subjected to a statistical test. A low *p*-value (*p*-value < 0.05) indicates that the results are statistically significant. * FDA: Food and Drug Administration. ** AAP: American Academy of Pediatrics.

**Table 3:** Respondents’ attitude towards the COVID-19 vaccination in children.

VAX Scale	*n* (%)	Negative Attitude	Age	Gender	Profession	Work Organization	Practice Area	Work Experience
Questions 1–3 (concerns about trusting vaccines)	297 (62.2)	Low	0.434	0.005 *	0.012 *	0.007 *	0.159	0.691
	118 (24.7)	Intermediate						
	62 (12.9)	High						
Questions 4–6 (concerns about unpredictable effects)	127 (26.6)	Low	0.667	0.46	0.004 *	0.352	0.342	<0.001 *
	233 (48.8)	Intermediate						
	117 (24.5)	High						
Questions 7–9 (concerns about commercial profits)	281 (58.9)	Low	0.112	0.001 *	0.776	0.081	0.153	0.452
	147 (30.8)	Intermediate						
	49 (10.2)	High						
Questions 10–12 (preference to natural immunity)	125 (26.)	Low	0.671	0.211	0.137	<0.001 *	0.486	0.091
	249 (52.2)	Intermediate						
	103 (21.5)	High						
Agreement with COVID-19 vaccines off-label use in children under 12 years of age	13 (2.7)	Low	0.66	0.891	0.305	0.017	0.723	0.164
	210 (44)	Intermediate						
	254 (53.2)	High						

* A *p*-value is a probability that is determined after data are subjected to a statistical test. A low *p*-value (*p*-value < 0.05) indicates that the results are statistically significant.

**Table 4:** Respondents’ perceptive towards off-label COVID-19 vaccination in children.

To What Extent Do You Agree or Disagree with the Following Statements?	Strongly Agree/Agree *n* (%)	Neutral *n* (%)	Disagree/Strongly Disagree *n* (%)
COVID-19 vaccines can be used off-label in children under 12	127 (26.6)	38 (7.9)	312 (65.4)
Off-label use of COVID-19 vaccines is an ethically permitted decision on a case-by-case basis	186 (38.9)	82 (17.1)	209 (43.8)
Off-label COVID-19 vaccination in children increases the probability of more rapid and effective protection against the SARS-CoV-2 virus	166 (34.8)	140 (29.3)	171 (35.8)
Off-label vaccination during a pandemic is an approach to protect patients after evaluating the associated risks and benefits	152 (31.8)	104 (21.8)	221 (46.3)
HCPs should consider prescribe/administer pediatric off-label COVID-19 vaccines	97 (20.3)	53 (11.1)	327 (68.5)
Pediatric off-label COVID-19 immunization is subject to the same legal and ethical standards as other cases of off-label usage	158 (33.1)	61 (12.7)	258 (54)
Administering vaccinations to minors under the age of 12 may result in consequences, including legal liability for providers	404 (84.6)	22 (4.6)	51 (10.6)
Emerging clinical studies will provide evidence on the most effective and safe dose and administration schedule for children	435 (91.1)	11 (2.3)	31 (6.4)

## Supplementary Materials

The following supporting information can be downloaded at: https://www.mdpi.com/article/10.3390/vaccines10081236/s1, Scheme 1: Pediatric off-label COVID-19 vaccination: the concerns of healthcare professionals in Pakistan.
