# Supplementary material for: Pediatric Off-Label COVID-19 Vaccination: The Concerns of Healthcare Professionals in Pakistan
Source: Vaccines (Basel). 2022 Aug 2;10(8):1236. doi: 10.3390/vaccines10081236 (PMC9414769; doi:10.3390/vaccines10081236)
Supplement: Supplementary file 1 [file vaccines-10-01236-s001.zip › vaccines-1793249-supplementary XML.pdf]

Scheme 1. Pediatric off-label COVID-19 vaccination: the concerns of healthcare professionals in Pakistan.

Dear Sir/Madam,
Thank you for agreeing to voluntarily participate in this study. This questionnaire aims to explore your awareness, knowledge and attitude about COVID-19 vaccination. There is no risk to participate since we are collecting only your viewpoints. Though, the study outcomes would deliver an enhanced understanding of your approach towards COVID-19 vaccination. This study would not provide any direct advantage and you will not get any compensation. Please be advised that all information will be dealt with in strict confidence and will be used for research purposes only.

Age:_________years **Gender:** (a) male (b) female
Health profession: a) Physician b) Pharmacist c) Dentist d) Nurse e) Other (please specify) _________
Organization: (a) private (b) government
Practice area: a) Primary patient care b) Secondary patient care c) Tertiary patient care
Year of experience: (a)Less than 1 year (b) 1-5 years (c)6-10 years (d)11-15 years (e) > 15 years

Knowledge about COVID-19 vaccines and its off-label use:

Q1. Do you know what Off-Label vaccination is? a) Yes b) No c) Don't know

Q2. Do you know any vaccine (other than COVID vaccine) which is administered off-label in children? a) Yes b) No c) Don't know (if yes, kindly mention _______________)

Q3. Do you know which COVID-19 vaccines have been approved for children?
a) Yes b) No c) Don't know

Q4. Do you know that COVID-19 vaccine is beneficial for children 5 to 11 years?
a) Yes b) No c) Don't know

Q5. Do you know that COVID-19 vaccine for children ages 5 through 11 is safe and effective?
a) Yes b) No c) Don't know

Q6. Do you know that the FDA and the American Academy of Pediatricians both caution against using the COVID-19 vaccine off-label in children under 12? a) Yes b) No c) Don't know

Q7. Do you know that children should receive the age-appropriate vaccine formulation regardless of their size or weight? a) Yes b) No c) Don't know

Q8**.**Do you know that there is a difference in formulation of the COVID-19 vaccines for children and adults? a) Yes b) No c) Don't know

Q9. Do you know that there is a difference in the dosing of the COVID-19 vaccines for children and adults? a) Yes b) No c) Don't know

Q10. Do you know how to monitor safety in vaccinated children? a) Yes b) No c) Don't know

Q11. Do you know that children can get other vaccines in combination with COVID-19 vaccine in the same visit? a) Yes b) No c) Don't know

Q12. Do you know that vaccine may have mild, self-limiting side effects which are normal signs their body is building protection? a) Yes b) No c) Don't know

Q13. Do you know that the severe reactions after vaccination are possible, but only in rare cases?
a) Yes b) No c) Don't know

Q14. Do you know that COVID-19 vaccines do not cause any developmental or fertility problems in vaccinating children? a) Yes b) No c) Don't know

Q15. Do you think that there is a lower risk for myocarditis and pericarditis in children as compared to adults after vaccination? a) Yes b) No c) Don't know

Q16. Do you know that children who have already had COVID-19 should still get the vaccine?
a) Yes b) No c) Don't know

Q17. Do you know that getting a vaccine can help protect children ages 5 years and older from spreading COVID-19 to others? a) Yes b) No c) Don't know

Q18. Do you know that vaccination can help keep children from getting seriously sick even if they do get COVID-19? a) Yes b) No c) Don't know

Attitude about COVID-19 vaccines using Vaccination Attitudes Examination (VAX) Scale

**Statement**	**Strongly agree**	**Agree**	**Neutral**	**Somewhat disagree**	**Disa-gree**	**Strongly disagree**
Do you think that COVID-19 vaccines are safe for children under 12 years of age?						
Can you rely on vaccines to stop serious consequences of COVID-19?						
Do you think that children are protected after getting vaccinated?						
Do you think that there may be problems associated with the use of vaccine that have not yet discovered?						
Do you believe that vaccines can cause unpredicted complications in children?						
Are you concern about the unidentified effects of vaccines in the future?						
Do you think that vaccines make a lot of money for pharmaceutical firms, but do not do much for regular people?						
Do you think that authorities promote vaccination for financial gain, not for publics' health?						
Do you think that vaccination programs are a big con?						
Do you think that natural immunity lasts longer than a vaccination?						
Do you think that natural exposure to COVID-19 gives the safest protection?						
Do you think that being exposed to COVID-19 naturally is safer for the immune system than being exposed through vaccination?						

**Respondents' perceptive towards the off-label COVID-19 vaccination in children**	**Strongly agree**	**Agree**	**Neutral**	**Disagree**	**Strongly disagree**
Do you think that COVID-19 vaccines can be used off-label in children under 12 years of age?					
Do you think that off-label use of COVID-19 vaccines is an ethically permissible option on a case-by-case basis?					

Do you think that off-label COVID-19 vaccination in children is the possibility of faster and more effective protection against the SARS-COV-2 virus?					
Do you think that off-label vaccination during pandemic is an approach to protect patients after evaluating associated risks and benefits?					
Do you think that healthcare providers should consider recommending or administering pediatric COVID-19 vaccines off-label?					
Do you think that pediatric off-label COVID-19 immunization is subject to the same legal and ethical standards as other cases of off-label usage?					
Do you think that administering vaccines off-label to children under 12 may result in consequences, including legal responsibility for providers?					
Do you believe that emerging clinical studies will provide evidence on the most effective and safe dose and administration schedule for children?					

Do you keep vigilance on official websites for COVID-19 updates?

a) Yes b) No

Your reliable source of information for COVID-19 updates?

a. Colleague/ peer b. Medical Journals/literature c. Electronic media

d. Ministry of health website e. Other

THANK YOU VERY MUCH INDEED FOR ASSISTING WITH THIS RESEARCH
